# Anti-Inflammatory Cembranoids from the Soft Coral *Lobophytum crassum*

**DOI:** 10.3390/md15100327

**Published:** 2017-10-23

**Authors:** Kuei-Hung Lai, Wan-Jing You, Chi-Chen Lin, Mohamed El-Shazly, Zuo-Jian Liao, Jui-Hsin Su

**Affiliations:** 1 National Museum of Marine Biology & Aquarium, Pingtung 94450, Taiwan; mos19880822@gmail.com (K.-H.L.); joy5852030@gmail.com (W.-J.Y.); liao771107@gmail.com (Z.-J.L.); 2 Graduate Institute of Marine Biology, National Dong Hwa University, Pingtung 94450, Taiwan; 3 Department of Life Sciences, Institute of Biomedical Science, National Chung Hsing University, Taichung 40249, Taiwan; lincc@dragon.nchu.edu.tw; 4 Department of Pharmacognosy and Natural Products Chemistry, Faculty of Pharmacy, Ain-Shams University, Organization of African Unity Street, Abassia, Cairo 11566, Egypt; mohamed.elshazly@pharma.asu.edu.eg; 5 Department of Pharmaceutical Biology, Faculty of Pharmacy and Biotechnology, German University in Cairo, Cairo 11432, Egypt; 6 Doctoral Degree Program in Marine Biotechnology, National Sun Yat-Sen University (NSYSU), 70 Lien-Hai Road, Kaohsiung 80424, Taiwan; 7 Doctoral Degree Program in Marine Biotechnology, Academia Sinica, 128 Academia Road, Section 2, Nankang, Taipei 11529, Taiwan

**Keywords:** lobophyolides, *α*-epoxylactone group, cembranoids, IL-12 production, NO release

## Abstract

Cembrane-type diterpenoids are among the most frequently encountered natural products from the soft corals of the genus *Lobophytum*. In the course of our investigation to identify anti-inflammatory constituents from a wild-type soft coral *Lobophytum crassum*, two new cembranoids, lobophyolide A (**1**) and B (**2**), along with five known compounds (**3**–**7**), were isolated. The structures of these natural products were identified using NMR and MS spectroscopic analyses. Compound **1** was found to possess the first identified *α*-epoxylactone group among all cembrane-type diterpenoids. The in vitro anti-inflammatory effect of compounds **1**–**5** was evaluated. The results showed that compounds **1**–**5** not only reduced IL-12 release, but also attenuated NO production in LPS-activated dendritic cells. Our data indicated that the isolated series of cembrane-type diterpenoids demonstrated interesting structural features and anti-inflammatory activity which could be further developed into therapeutic entities.

## 1. Introduction

Soft corals, which belong to the order Alcyonacea, are a group of corals missing a calcium carbonate skeleton. They usually grow on the hard surfaces of benthonic animals exposing their soft tissue. Therefore, these corals develop special chemical defense mechanisms and produce a large variety of secondary metabolites against predators, bacteria, and parasites, promoting their own protection and survival [1]. These secondary metabolites were found to possess a plethora of interesting biological activities at low concentrations, suggesting their potential application as therapeutic agents. 

**Figure 1:**
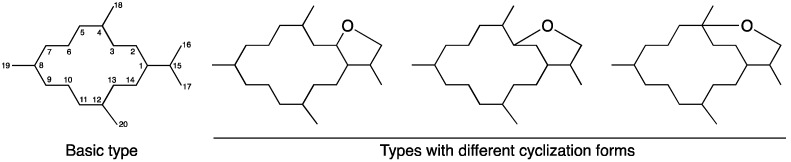
Structural characteristics of cembrane-type diterpenoids.

The soft corals of genus *Lobophytum* have been studied for decades since the first compound, lobophytolide, a cembrane-type diterpenoid, was identified from *Lobophytum cristagalli* [2]. Further investigation of this genus revealed more than 250 compounds categorized into cembrane-type diterpenoids [2,3,4,5,6,7,8,9,10,11,12,13,14,15,16,17,18,19,20,21,22,23,24,25,26,27,28,29,30,31,32,33,34,35,36,37,38,39,40,41,42,43,44,45,46,47,48,49,50,51,52,53,54,55,56,57,58], other types of diterpenoids [59,60,61,62,63,64,65], lipids [66,67,68,69], steroids [44,69,70,71,72,73,74,75,76,77,78], tocopherols [79], triterpenoids [80], and zoanthamine-type alkaloids [81]. Our group has extensively studied cembrane-type diterpenoids over the past few years and has found that they demonstrate a wide structural diversity [82]. This class of compounds possesses a three-methyl substituted 14-memberd ring system with an isopropyl unit, whereas this isopropyl moiety can be replaced with several types of side rings including a *γ*-lactone or an unsaturated five-membered ring, a *δ*-lactone or an unsaturated six-membered ring, and an *ɛ*-lactone or an unsaturated seven-membered ring (Figure 1). Moreover, the structural changes such as epoxidation, allylic, and isopropyl oxidation or cyclization can be found in almost all unsaturated parts of the ring system [5,8,11].

The pharmacological properties of cembranoids were extensively studied with special emphasis on their anti-inflammatory activity. Their effect was evaluated on iNOS (inducible nitric oxide synthase) [10,13], COX-2 (cyclooxygenase-2) [10,19], NF-*κ*B (nuclear factor kappa-light-chain-enhancer of activated B cells) [19,20], and neutrophil elastase release [15,18], as well as superoxide anion [15] and NO reduction [12,23]. Additionally, these compounds showed potent antiviral [31,49] and anti-bacterial activities [40,43], as well as brine shrimp [45] and fish toxicity [45]. The structural diversity and interesting biological activity of cembrane-type diterpenoids encouraged us to search for other members of this interesting compound from the soft corals of the genus *Lobophytum*. We isolated seven cembranoids from a wild-type soft coral *Lobophytum crassum*, including two new and five known compounds. Compound **1** demonstrated a unique *α*-epoxylactone group which has never been found as a side ring of cembrane-type diterpenoids. Compound **6** showed a 16-acetyl substitution, rendering it the first member of this group with such a structural feature to be isolated from natural sources. The anti-inflammatory activity of compounds **1**–**5** was evaluated using in vitro models for detecting LPS-induced interleukin 12 (IL-12) release and nitric oxide (NO) production in dendritic cells. 

## 2. Results

### 2.1. Chemical Identification of Cembranoids

**Figure 2:**
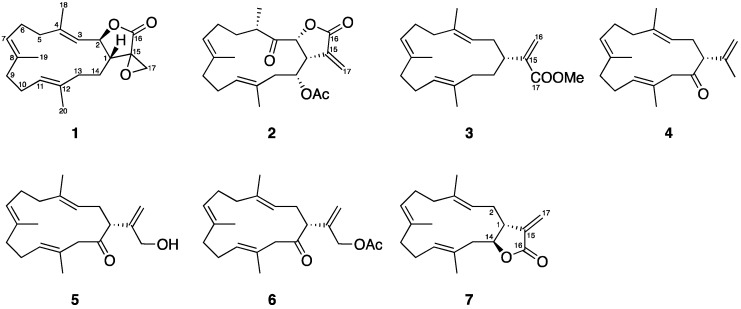
Cembranoids isolated from the soft coral *Lobophytum crassum*.

The EtOAc extract of the freeze-dried specimen of the soft coral *Lobophytum crassum* was chromatographed using normal phase silica gel and the eluting fractions were further separated and purified utilizing reversed phase HPLC to yield **1**–**7** (Figure 2). The new compounds were named as lobophyolide A (**1**) and B (**2**) and the known compounds were identified as 16-methoxycarbonyl-cembrene A (**3**) [50], sinarone (**4**) [83], sinulariol D (**5**) [83], 16-acetyl-sinulariol D (**6**) [83], and (*E*,*E*,*E*)-6,10,14-trimethy-3-methylene-trans-3*α*,4,7,8,11,12,15,15*α*-octahydrocy clotetradeca[*β*]furan-2(3H)-one (**7**) [3].

**Figure 3:**
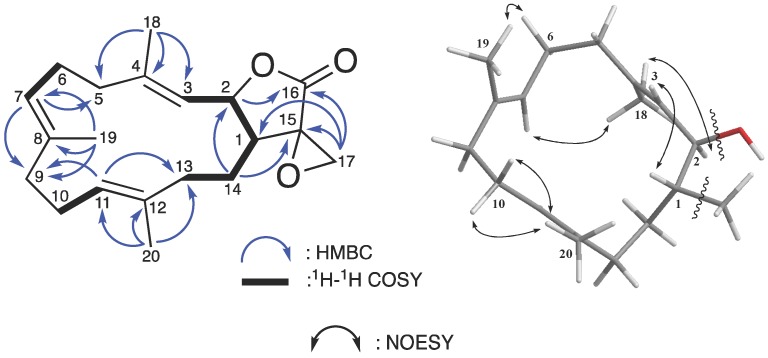
Selective ^1^H–^1^H COSY, HMBC, and NOESY correlations of **1**.

Compound **1** was isolated as a colorless oil. The analysis of its ^13^C NMR and HRESIMS data deduced its molecular formula, C_20_H_28_O_3_, which suggested seven degrees of unsaturation. The ^1^H and ^13^C NMR data (Table 1) demonstrated the signals of twenty-eight protons and twenty carbons sorted by DEPT and HSQC spectra. An ester carbonyl group (*δ*_C_ 173.8) was detected along with three pairs of C=C double bonds [*δ*_C_/*δ*_H_ 142.5 (C-4), 133.8 (C-8), 131.4 (C-12), 125.2 (C-11)/4.93 (H-11, t, *J* = 5.0 Hz), 125.1 (C-7)/4.89 (H-7, t, *J* = 5.0 Hz), and 122.7 (C-3)/5.17 (H-3, d, *J* = 10.0 Hz)], an oxymethine [*δ*_C_/*δ*_H_ 79.4 (C-2)/5.06 (H-2, dd, *J* = 10.0, 4.5 Hz)], an oxymethylene [*δ*_C_ 52.2 (C-17)/2.96 (H-17, d, *J* = 6.0 Hz), 3.30 (H-17, d, *J* = 6.0 Hz)], a sp^3^ quaternary carbon [*δ*_C_ 57.9 (C-15)], and three tertiary methyls [*δ*_H_ 1.52 (H-20, s), 1.59 (H-19, s), and 1.74 (H-18, s)]. Detailed analysis of these NMR data revealed three individual methyl-bearing trisubstituted double bond moieties which suggested the presence of a typical cembranoid skeleton (a three-methyl substituted 14-membered ring system) [84]. This partial structure was further confirmed by the HMBC cross-peaks from H-18 to C-3, C-4, and C-5; from H-19 to C-7, C-8, and C-9; from H-20 to C-11, C-12, and C-13; from H-3 and H-7 to C-5; from H-7 and H-11 to C-9; and from H-14 to C-2 (Table 1) (Figure 3). 

Excluding the four unsaturated groups from the main cembranoid structure, the additional three unsaturated degrees were attributed to an unsaturated ring system supported by the observation of a unique *α*-epoxylactone chemical shift pattern from the ^13^C NMR spectra (Table 1) [85]. Moreover, the IR spectrum showed characteristic absorptions at 927 cm^−1^ and 1785 cm^−1^, suggesting the presence of an epoxide [86] and a *γ*-lactone moiety [87]. The HMBC cross-correlations (from H-17 to C-1; and from H-14 to C-2 and C-15) connected these two units and the ^1^H–^1^H COSY and HMBC (from H-17 to C-15 and C-16) further revealed that 1 possessed one 1,1-disubstituted epoxide at C-17 (Figure 3), which implied a five-membered side ring cembranoidal diterpene derivative.

Assuming a *β*-oriented H-1 (*δ*_H_ 2.26 m) in compound **1**, the relative configuration was determined by studying NOESY spectra (Figure 3). The presence of the COSY but not NOESY correlations between H-1 and H-2 indicated a *trans*-fused lactone with *α*-oriented H-2, which was also supported by the proton vicinal coupling constant of H-1/H-2 (*J* = 4.5 Hz) [88]. In this type of cembranoid, the *E*-form C=C double bond systems are usually found at C-3/C-4 and C-7/C-8 and sometimes at C-11/C-12 [23]. The missing NOESY correlations between H-20/H-11, H-19/H-7, and H-18/H-3 implied an *E*-configuration for these three C=C double bonds [84]. The up-fielded ^13^C NMR chemical shift of C-20 (*δ*_C_ 15.9) supported such a proposal because it differed from the previously reported *Z*-form olefinic methyl group (*δ*_C_ 20.1) [26]. According to the above data, the relative configuration of **1** was suggested as 1R*, 2R*, 3*E*, 7*E*, and 11*E* and the compound was named as lobophyolide A.

**Figure 4:**
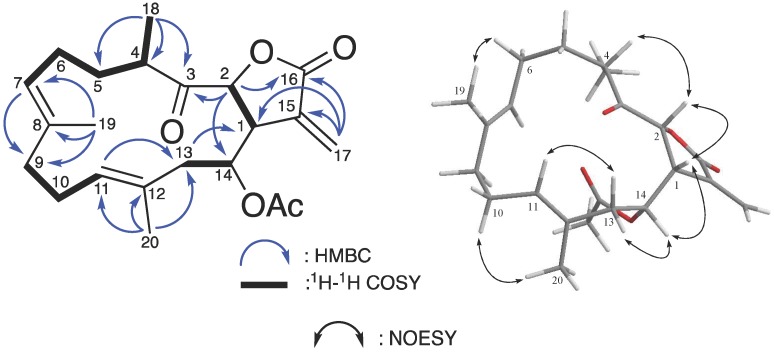
Selective ^1^H–^1^H COSY, HMBC, and NOESY correlations of **2**.

Compound **2**, was obtained as colorless oil through the same procedure. The molecular formula (C_22_H_30_O_5_) was inferred from HRESIMS and ^13^C NMR data (Table 2), indicating eight degrees of unsaturation. The IR spectrum demonstrated the presence of several C=O functional groups based on the absorbed peaks at 1778, 1742, and 1719 cm^−1^. The ^1^H, ^13^C, and HSQC NMR revealed the presence of a ketone carbonyl carbon (*δ*_C_ 209.9), two ester carbonyl carbons (*δ*_C_ 170.0 and *δ*_C_ 169.5), three pairs of C=C double bonds [*δ*_C_/*δ*_H_ 135.2 (C-8), 135.1 (C-15), 130.3 (C-11)/5.20 (H-11, t, *J* = 6.3 Hz), 129.3 (C-12), 125.7 (C-7)/4.98 (H-7, m), and 124.8 (C-3)/5.73 (H_b_-3, d, *J* = 2.5 Hz), 6.42 (H_a_-3, d, *J* = 2.5 Hz)], two oxymethines [*δ*_C_/*δ*_H_ 80.2 (C-2)/4.97 (H-2, d, *J* = 3.5 Hz), 75.6 (C-14)/5.09 (H-14, dt, *J* = 11.0, 2.5 Hz)], three tertiary methyls [*δ*_H_ 2.02 (H-14-OAc, s), 1.72 (H-20, s), and 1.49 (H-19, s)], and a secondary methyl at *δ*_H_ 1.14 (H-18, d, *J* = 7.0 Hz) (Table 2). Detailed analysis of these NMR data suggested that **2** also displayed a type IIa cembranoid skeleton and was closely related to a previously reported compound, (1*S*,2*S*,3*E*,7*E*,11*E*)-3,7,11,15-cembratetraen-17,2-olide [80], with a missing C=C double bond moiety, as well as additional groups of a ketone carbonyl (*δ*_C_ 209.9) and an acetyl [*δ*_C_/*δ*_H_ 170.0, 21.0/2.02 (s)]. According to the HMBC cross-peaks (from H-18 and H-2 to C-3; from H-2 to C-14), two additional oxygen bearing groups were added into the main 14-membered ring system, a ketone group at C-3 and an acetyl group at C-14 (Figure 4).

The relative configuration of the four chiral centers in compound **2** was suggested based on the analysis of the NOESY data assuming a *β*-orientation of H-1 [*δ*_H_ 3.26 dd (3.0, 2.0)] (Figure 4). Since H-2 was correlated to H-1 and H-4 in the NOESY spectrum, the *β*- and *α*-orientations of H-2 and H-18 were suggested, respectively. The *α*-orientation of the 14-acetyl group was proposed based on the H-1/H-14 NOESY correlation. Moreover, the observation of the NOESY cross-peaks between H-6/H-19 and H-10/H-20 suggested an *E* configuration of these two C=C double bond systems (C-7/C-8 and C-11/C-12). Hence, the relative configuration was elucidated as 1R*, 2R*, 4S*, 14R*, 7*E*, and 11*E*, and the structure of compound **2** was established and named as lobophyolide B.

### 2.2. Anti-Inflammatory Activity of the Isolated Cembranoids

The anti-inflammatory activity of compounds **1**–**5** was evaluated using the in vitro models for the detection of LPS-induced interleukin 12 (IL-12) release and nitric oxide (NO) production in dendritic cells (Table 3). The survival rate of the treated dendritic cells was also tested to determine the cytotoxicity of the isolates. The results showed that compounds **1**–**3** and **5** (below 50 μg/mL) exhibited a potent inhibitory effect against cytokine IL-12 and NO production with inhibition rates ranging from 86.1% to 96.2%, but also showed considerable cytotoxicity. Compound **1** was found to be the best lead showing potent activity with minimum toxicity compared with the other tested cembranoids. These results suggested that further semisynthetic modifications are necessary for the introduction of a safe and potent anti-inflammatory cembranoid. 

## 3. Material and Methods

### 3.1. General Experimental Procedure

Optical rotation spectra were recorded on a JASCO P-1010 polarimeter (JASCO, Tokyo, Japan). IR spectra were obtained on a Fourier-transform IR spectrophotometer Varian Digilab FTS 1000 (Varian Inc., Palo Alto, CA, USA). NMR spectra were detected on a Varian Unity INOVA 500 FT-NMR instrument (Varian Inc., Palo Alto, CA, USA). High resolution electrospray ionization mass spectrometry (HRESIMS) analyses were performed on a Bruker APEX II instrument (Bruker Daltonik, Bremen, Germany). Gravity column chromatography was performed with 230–400 mesh silica gel (Merck, Darmstadt, Germany). TLC was performed on 0.25 mm thick precoated Kieselgel 60 F254 (Merck, Darmstadt, Germany) and/or 0.25 mm RP-18 F_254S_ (Merck, Darmstadt, Germany) coated plates and was then visualized by spraying with 10% H_2_SO_4_ and heating on a hot plate. A Hitachi L-7100 pump, Rheodyne 7725 injection port, and a Hitachi L-2455 Photodiode Array Detector (Hitachi, Tokyo, Japan), along with a preparative normal phase column Supelco Ascentis^®^ Si Cat #: 581514-U (25 cm × 10 mm, C18, 5 μm) and a reverse phase column Supelco Ascentis^®^ C-18 Cat #: 581343-U, were used for RP-HPLC. All methods were carried out in accordance with the relevant guidelines and regulations.

### 3.2. Animal Material

Specimens of wild soft coral of *Lobophytum crassum* were collected by scuba diving at a depth of around 8 m off the coast of Pingtung, Taiwan (specimen No. 2016-11-14-SP). A voucher specimen was deposited in the National Museum of Marine Biology and Aquarium, Pingtung, Taiwan.

### 3.3. Extraction and Isolation

The wild soft coral of *Lobophytum crassum* (830 g, wet weight) was freeze-dried, and the resulting dry material (290 g) was then extracted exhaustively with EtOAc. The EtOAc extract was evaporated under reduced pressure to afford a residue (13.6 g). The residue was subjected to column chromatography on silica gel, using the mixtures of *n*-hexane, EtOAc, and acetone with increasing polarity (*n*-hexane:EtOAc:acetone, 50:1:0, 30:1:0, 20:1:0, 10:1:0, 8:1:0, 5:1:0, 3:1:0, 1:1:0, 1:2:0, 0:1:0, and 0:0:1) to yield 11 fractions. Fr-6 (3.7 g) was fractioned with LH-20 eluting with 100% acetone to afford eight subfractions (6-L1–6-L8). Fr-6-L5 and Fr-6-L6 were combined (540 mg) and then separated by normal-phase HPLC (*n*-hexane:acetone 3:1) to gain 19 more subfractions (6-L5-1–6-L5-19). Subfraction 6-L5-3 (2.7 mg) was purified by reversed-phase HPLC (90% MeOH) to afford **3** (0.5 mg) and **4** (0.7 mg). Subfractions 6-L5-4–6-L5-6 were combined (40.9 mg) and purified by reversed-phase HPLC (90% MeOH) to afford **1** (0.8 mg) and **7** (8.5 mg). Subfraction 6-L5-9 (34.3 mg) was also purified by reversed-phase HPLC (70% acetonitrile) to afford **2** (3.4 mg). Subfraction 6-L5-10 (44.5 mg) was purified by normal-phase HPLC (hexane:EtOAc:dichloromethane 8:1:1) to afford **5** (4.2 mg). Compound **6** (1.5 mg) was obtained from subfractions 6-L5-11–6-L5-13 (35.0 mg) using normal-phase HPLC (n-hexane:EtOAc 7:1).

Lobophyolide A (**1**): colorless oil; ${\left[\alpha \right]}_{D}^{21}$ = −55.2 (*c* 0.1000, CHCl_3_); IR (neat, CHCl_3_) *ν*_max_ 2921, 2852, 1785, 1441, 1385, 1314, 1295 cm^−1^; ^13^C (CDCl_3_, 125 MHz) and ^1^H (CDCl_3_, 500 MHz) NMR data, see Table 1; ESIMS *m*/*z* 339 [M + Na]^+^; HRESIMS *m*/*z* 339.19301 [M + Na]^+^ (calcd for C_20_H_28_O_3_Na, 339.19307).

Lobophyolide B (**2**): colorless oil; ${\left[\alpha \right]}_{D}^{20}$ = +1.2 (*c* 0.0750, CHCl_3_); IR (neat, CHCl_3_) *ν*_max_ 2923, 2854, 1778, 1742, 1719, 1437, 1374, 1271, 1228 cm^−1^; ^13^C (CDCl_3_, 125 MHz) and ^1^H (CDCl_3_, 500 MHz) NMR data, see Table 2; ESIMS *m*/*z* 397 [M + Na]^+^; HRESIMS *m*/*z* 397.19837 [M + Na]^+^ (calcd for C_22_H_30_O_5_Na, 327.22945).

16-Acetyl-sinulariol D (**6**): colorless oil; ${\left[\alpha \right]}_{D}^{21.3}$ = +7.5 (*c* 0.0375, CHCl_3_); IR (neat, CHCl_3_) *ν*_max_ 2923, 1778, 1651, 1436, 1373, 1227 cm^−1^; ^13^C (CDCl_3_, 125 MHz) and ^1^H (CDCl_3_, 500 MHz) NMR data, see supporting information; ESIMS *m*/*z* 353 [M + Na]^+^; HRESIMS *m*/*z* 353.24514 [M + Na]^+^ (calcd for C_22_H_34_O_2_Na, 353.24510).

### 3.4. Preparation of Mouse Bone Marrow-Derived Dendritic Cells (DCs)

C57BL/6 mice, which were purchased from Taiwan, were used in this study. All animals were housed in a specific pathogen-free facility in the Division of Laboratory Animals, China Medical University. All mice were maintained and handled according to standard protocols and the protocols were approved (103-156-N, 27 December 2012) by the Institutional Animal Care and Use Committee, China Medical University. The bones of mice were collected and bone marrow-derived dendritic cells (DCs) were prepared as previously described [89]. In brief, bone marrow cells were isolated from femurs and tibias and seeded on 24-well culture plates (Corning, New York, NY, USA) in 1 mL/well complete RPMI 1640 medium (Gibco, Waltham, MA, USA), and 10 ng/mL recombinant mouse GMCSF (Peprotech, Rocky Hill, NJ, USA). At day three, fresh medium (1 mL/well) containing 10 ng/mL GM-CSF was added. At day five, half of the cell-free supernatant was exchanged and fresh medium containing 10 ng/mL GMCSF was added. The seven-day-cultured DCs (>80% CD11c^+^ cells) were used for all experiments. In the T cell activation experiment, DCs were purified by an EasySep Positive Selection Kit (StemCell Technology, Vancouver, BC, Canada) according to the manufacturer’s instructions. The purity of CD11c^+^ cells was over 95%. The OT-II TCR-transgenic mice were provided by Dr. Clifford Lowell (UCSF, San Francisco, CA, USA). All animals were kept in a specific pathogen-free facility (NHRI, Miaoli, Taiwan) and handled according to protocols approved by the Institutional Animal Care and Use Committee of NHRI.

### 3.5. Measurement of Cytokines Production by DCs

Cytokine production was measured by an enzyme-linked immunosorbent assay (ELISA) as described previously [89]. The DCs were treated with lipopolysaccharide (LPS, 100 ng/mL) from *Escherichia coli* 055:B5 (Sigma, St. Louis, MO, USA) or LPS+testing cembranoidal compounds for 24 h for IL-12. The production of IL-12 was measured at 570 nm using the ELISA kit (eBioscience, San Diego, CA, USA).

### 3.6. Measurement of Nitric Oxide (NO) Production by DCs

The supernatants were collected from DCs (1 × 10^6^ /mL) propagated in the presence or absence of testing cembranoidal compounds for 1 h. The cells were then stimulated with 100 ng/mL LPS. A total of 100 µL of cell culture supernatant was reacted with 100 µL of Griess reagent (1:1 mixture of 0.2% *N*-(1-naphthyl) ethylenediamine dihydrochloride in water and 2% sulfanilamide) in a 96-well plate and incubated at room temperature for 10 min. The absorbance at 540 nm was recorded using sandwich ELISA assays, according to the manufacturer’s specifications (PeproTech) [89]. Fresh medium was used as the blank. The results are expressed as the percentage of inhibition calculated relative to the cells treated with vehicle and LPS.

### 3.7. Cell Viability Assay

The cytotoxicity of the tested cembranoids (Sigma, St. Louis, MO, USA) was measured by Cell Counting Kit 8 (CCK-8 Dojindo Laboratories, Kumamoto, Japan) according to the manufacturer’s instructions. In brief, 100 μL of 5 × 10^3^ DCs was plated into each well of a 96-well plate and incubated overnight, and cells were then treated with various concentrations of naringenin (dissolved in DMSO and made a stock solution at 400 mM; Sigma-Aldrich, St. Louis, MO, USA) for an additional 24 h. Subsequently, 10 μL of the CCK-8 solution was added to each well and incubated at 37 °C for 3 h. The absorbance was recorded on a microplate reader (Tecan, Durham, NC, USA) at the wavelength of 450 nm.

### 3.8. Statistics

The results were expressed as the mean ± standard deviation (SD). Comparison in each experiment was performed using an unpaired Student’s *t*-test and a *p* value of less than 0.05 was considered to be statistically significant.

## 4. Conclusions

A series of cembrane-type diterpenoids were isolated from the ethyl extract of a wild-type soft coral *Lobophytum crassum*. Two new cembranoids, lobophyolide A (**1**) and B (**2**), as well as five known compounds (**3**–**7**), were identified. Compound **1** demonstrated a unique structural feature among all cembrane-type diterpenoids with an *α*-epoxylactone group, which might be derived from the terminal double bond moiety of (1*S*,14*S*)-(*E*,*E*,*E*)-15-methylene-3,7,11-trimethyl-17-oxabicyclo<12.3.0^1,14^>heptadeca-2,6,10-trien-16-one [90,91]. The anti-inflammatory effect of compounds **1**–**5** was examined. Among all tested compounds, **1** showed the highest therapeutic index with less toxicity and potent activity in inhibiting IL-12 release and attenuating NO production from LPS-activated dendritic cells. These results suggest the potential application of this class of compounds as anti-inflammatory agents, especially with the recent advances in culture protocols for this soft coral developed by our group, as well as the success in the total synthesis of cembrane-type diterpenoids (7-acetylsinumaximol B) [92], which could supply necessary quantities of active cembrane-type diterpenoids for industrial development. 

## Figures and Tables

**Table 1:** ^1^H, ^13^C, ^1^H–^1^H COSY, and HMBC NMR data of **1**.

Position	*δ*_H_ (*J* in Hz) *^a^*	*δ*_C_ (Mult.) *^b^*	^1^H–^1^H COSY	HMBC
1	2.26 m	39.0 (CH)	H-2, H-14	
2	5.06 dd (10.0, 4.5)	79.4 (CH)	H-1, H-3	C-16
3	5.17 d (10.0)	122.7 (CH)		C-5
4		142.5 (C)		
5	2.22 m	38.4 (CH_2_)		
6	2.21 m; 2.30 m	24.1 (CH_2_)	H-7	
7	4.89 t (5.0)	125.1 (CH)	H-6	C-5, C-9
8		133.8 (C)		
9	1.99 m; 2.12 m	39.0 (CH_2_)		
10	2.07 m; 2.20 m	23.9 (CH_2_)	H-11	
11	4.93 t (5.0)	125.2 (CH)	H-10	C-9, C-13
12		131.4 (C)		
13	2.03 m	35.1 (CH_2_)	H-14	
14	1.55 m; 1.77 m	24.2 (CH_2_)	H-13, H-1	C-2, C-15
15		57.9 (C)		
16		173.8 (C)		
17	2.96 d (6.0); 3.30 d (6.0)	52.2 (CH_2_)		C-1, C-15, C-16
18	1.74 s	16.4 (CH_3_)		C-3, C-4, C-5
19	1.59 s	15.2 (CH_3_)		C-7, C-8, C-9
20	1.52 s	15.9 (CH_3_)		C-11, C-12, C-13

Spectra recorded at *^a^* 500 and *^b^* 125 MHz in CDCl_3_.

**Table 2:** ^1^H, ^13^C, ^1^H–^1^H COSY, and HMBC NMR data of **2**.

Position	*δ*_H_ (*J* in Hz) *^a^*	*δ*_C_ (Mult.) *^b^*	^1^H–^1^H COSY	HMBC
1	3.26 dd (3.0, 2.0)	41.9 (CH)	H-2	
2	4.97 d (3.5)	80.2 (CH)	H-1	C-3, C-14
3		209.9 (C)		
4	2.66 m	41.8 (CH)	H-5, H-18	
5	1.46 m; 1.95 m	31.3 (CH_2_)	H-4, H-6	
6	1.83 m; 2.20 m	26.0 (CH_2_)	H-5, H-7	
7	4.98 m	125.7 (CH)	H-6	C-9
8		135.2 (C)		
9	2.04 m; 2.18 m	39.3 (CH_2_)	H-9, H-11	
10	2.13 m; 2.24 m	24.4 (CH_2_)	H-11	
11	5.20 t (6.3)	130.3 (CH)	H-10	C-13
12		129.3 (C)		
13	2.31 d (13.0); 2.45 dd (15.0, 11.0)	41.1 (CH_2_)	H-14	C-1
14	5.09 dt (11.0, 2.5)	75.6 (CH)	H-13	
15		135.1 (C)		
16		169.5 (C)		
17	5.73 d (2.5); 6.42 d (2.5)	124.8 (CH_2_)		C-1, C-15, C-16
18	1.14 d (7.0)	17.7 (CH_3_)		C-3, C-4, C-5
19	1.49 s	15.0 (CH_3_)		C-7, C-8, C-9
20	1.72 s	16.1 (CH_3_)		C-11, C-12, C-13
14-OAC	2.02 s	21.0 (CH_3_)		C-14-OAc
		170.0 (C)		

Spectra recorded at *^a^* 500 and *^b^* 125 MHz in CDCl_3_.

**Table 3:** Inhibition of LPS-induced IL-12 release and NO production in dendritic cells.

Compounds	LPS-Induced IL-12 Release	LPS-Induced NO Production	Survivals of DCs
	(Inh%) *^a^*	(Inh%) *^a^*	(Survival%) *^b^*
1	93.4 ± 0.5	93.5 ± 6.5	76.0 ± 0.01
2	93.6 ± 0.0	95.9 ± 3.2	52.0 ± 0.04
3	86.3 ± 1.1	86.1 ± 2.2	75.0 ± 0.01
4	77.0 ± 1.5	54.9 ± 0.0	85.0 ± 0.08
5	92.6 ± 0.6	96.2 ± 2.2	51.0 ± 0.01
Quercetin *^c^*	86.4 ± 0.0	86.1 ± 3.0	85.0 ± 5.00

*^a^* Percentage of inhibition (Inh%) under the concentration of 50 μg/mL; *^b^* Survival percentage (Survival%) of DCs under the concentration of 50 μg/mL; *^c^* Positive control under the concentration of 50 μM.
